# Genome editing without nucleases confers proliferative advantage to edited hepatocytes and corrects Wilson disease

**DOI:** 10.1172/jci.insight.171281

**Published:** 2023-11-08

**Authors:** Agnese Padula, Michele Spinelli, Edoardo Nusco, Xabier Bujanda Cundin, Filomena Capolongo, Severo Campione, Claudia Perna, Amy Bastille, Megan Ericson, Chih-Chieh Wang, Shengwen Zhang, Angela Amoresano, Mariana Nacht, Pasquale Piccolo

**Affiliations:** 1 Telethon Institute of Genetics and Medicine, Pozzuoli, Italy.; 2 Department of Chemical Sciences, University of Naples Federico II, Naples, Italy.; 3 Pathology Unit, Cardarelli Hospital, Naples, Italy.; 4 LogicBio Therapeutics, Lexington, Massachusetts, USA.

**Keywords:** Genetics, Hepatology, Gene therapy, Monogenic diseases

## Abstract

Application of classic liver-directed gene replacement strategies is limited in genetic diseases characterized by liver injury due to hepatocyte proliferation, resulting in decline of therapeutic transgene expression and potential genotoxic risk. Wilson disease (WD) is a life-threatening autosomal disorder of copper homeostasis caused by pathogenic variants in copper transporter ATP7B and characterized by toxic copper accumulation, resulting in severe liver and brain diseases. Genome editing holds promise for the treatment of WD; nevertheless, to rescue copper homeostasis, ATP7B function must be restored in at least 25% of the hepatocytes, which surpasses by far genome-editing correction rates. We applied a liver-directed, nuclease-free genome editing approach, based on adeno-associated viral vector–mediated (AAV-mediated) targeted integration of a promoterless mini-*ATP7B* cDNA into the albumin (*Alb*) locus. Administration of AAV-Alb–mini-ATP7B in 2 WD mouse models resulted in extensive liver repopulation by genome-edited hepatocytes holding a proliferative advantage over nonedited ones, and ameliorated liver injury and copper metabolism. Furthermore, combination of genome editing with a copper chelator, currently used for WD treatment, achieved greater disease improvement compared with chelation therapy alone. Nuclease-free genome editing provided therapeutic efficacy and may represent a safer and longer-lasting alternative to classic gene replacement strategies for WD.

## Graphical abstract

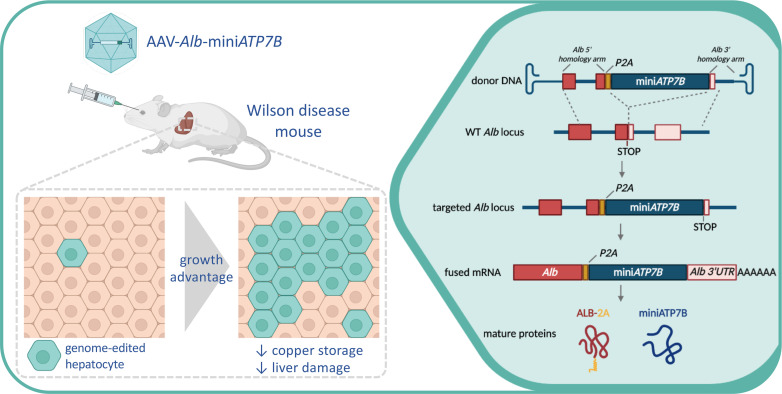

## Introduction

Wilson disease (WD) is a rare autosomal recessive disorder of copper metabolism caused by pathogenic variants in the *ATP7B* gene, which encodes a P-type copper-transporting ATPase and mainly is expressed in hepatocytes. ATP7B plays a critical role in copper metabolism, providing copper for cuproprotein synthesis and releasing excessive copper into the bile. Loss of ATP7B function results in toxic copper deposits in the liver and, to a lesser extent, in the brain, eyes, and kidneys, leading to chronic hepatitis and cirrhosis until liver failure, and to psychiatric and neurological deficits. Current therapies for WD are based on removal of copper deposits by chelating agents and reduction of copper intestinal absorption by zinc salts (1). Therapy is not effective in all patients with WD, and nonresponders usually require liver transplantation (2). Moreover, compliance with treatments is often an issue, especially among adolescents (3, 4).

Adeno-associated viral (AAV) vectors are considered the vector of choice for liver-directed gene therapy and are rapidly moving into the clinic (5). Classic gene replacement approaches using AAV vectors have achieved disease correction in adult *Atp7b^–/–^* mice (6). However, WD can manifest in young individuals, and early administration of episomal AAV vectors in growing livers may result in progressive loss of transgene expression due to hepatocyte proliferation. Moreover, most patients with WD present with liver damage already at the time of diagnosis (7), and the regenerative response may further promote transgene dilution. In contrast, genome editing results in permanent genomic DNA modifications that are inherited by daughter cells if proliferation occurs, thus avoiding transgene dilution.

AAV-mediated targeted integration of a promoterless transgene within the albumin (*Alb*) locus has been developed as a safe and effective liver-directed genome-editing approach (8). This strategy exploits homology-directed repair (HDR) to integrate a cDNA preceded by a sequence encoding for porcine teschovirus-1 2A peptide (P2A) at the 3′ end of the *Alb* gene coding sequence. Although the albumin promoter ensures robust liver-specific expression, thanks to P2A-driven ribosomal skipping the therapeutic transgene and albumin are expressed as separate proteins, and the secreted albumin protein is tagged with the 2A peptide, providing a circulating biomarker for site-specific integration and expression. Devoid of any promoter sequence, integrated promoterless vectors are not expected to transactivate neighboring genes and, thus, they are potentially safer in terms of genotoxicity. Spontaneous HDR is rather inefficient and results in on-target integration into, at most, 1% of the hepatocytes in healthy livers (8). However, we hypothesize that in the context of WD, genome editing–corrected hepatocytes cleared of the toxic copper may hold a proliferative advantage over noncorrected ones and can repopulate the liver, achieving significant therapeutic benefit.

## Results

### Integration of promoterless mini-ATP7B at the Alb locus conferred proliferative advantage, increased survival, and reduced copper storage in a lethal WD model.

We designed an AAV8 vector bearing a codon-optimized, human, mini-*ATP7B* cDNA devoid of sequences encoding for the first 4 metal binding domains (6, 9), flanked by 2 mouse *Alb* homology arms (HAs) and preceded by a P2A sequence (AAV8-Alb-ATP7BΔ1-4). Because HDR success rate depends upon HA size (10), we used an *ATP7B*Δ*1-4* cDNA to maximize HA size, which was 650 bp in length within our vector. At HDR targeted alleles, albumin will be expressed fused to P2A (ALB-2A), whereas a single proline residue from P2A will be translated at the N-terminus of mini-ATP7B (Supplemental Figure 1; supplemental material available online with this article; https://doi.org/10.1172/jci.insight.171281DS1).

**Figure 1:**
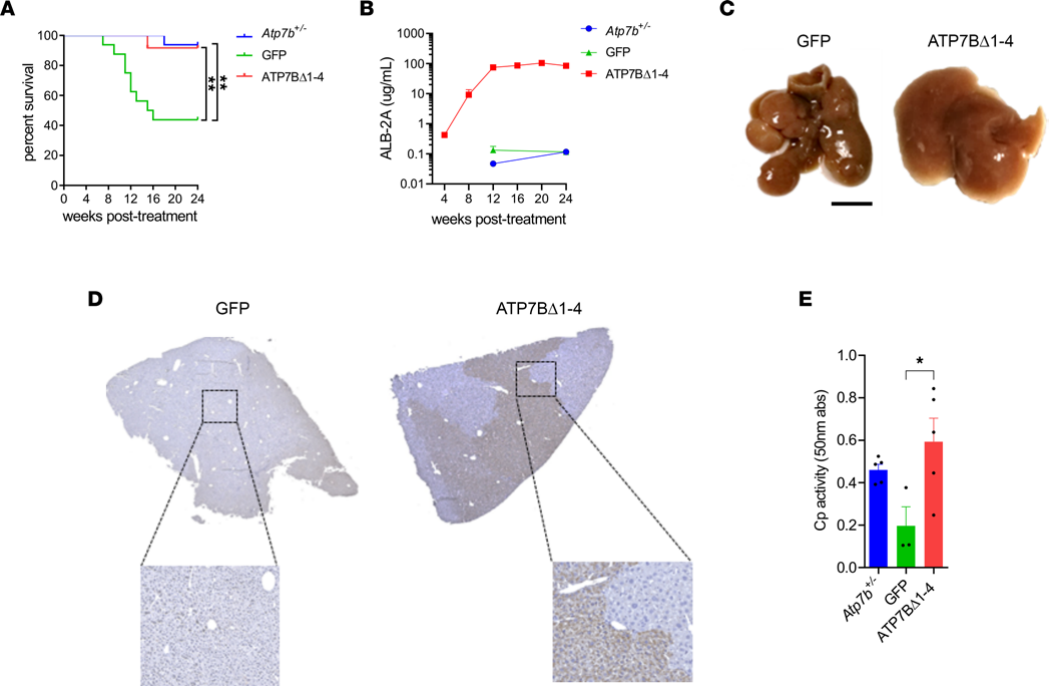
Promoterless mini-ATP7B rescues survival and confers proliferative advantage to corrected hepatocytes. *Atp7b^–/–^* mice (6 weeks old) were injected with 2.3 × 10^13^ gc/kg AAV8-GFP (GFP; *n* = 17) or AAV8-Alb-ATP7BΔ1-4 (ATP7BΔ1-4; *n* = 12). *Atp7b^+/-^* mice (*n* = 17) are shown as heathy controls. (**A**) Survival curves. For log-rank test, **P < 0.01. (**B**) Serum levels of ALB-2A fusion protein. (**C**) Representative photographs of dissected livers. Scale bar: 1 cm. (**D**) Representative images from liver IHC using anti–ATP7B Ab. (**E**) Serum ceruloplamin (Cp) activity. For 1-way ANOVA plus Tukey’s post hoc test, *P < 0.05.

To evaluate the efficacy of the AAV8-Alb-ATP7BΔ1-4 vector, 6-week-old *Atp7b^–/–^* mice were injected i.v. with 2.3 × 10^13^ genome copies (gc)/kg AAV8-Alb-ATP7BΔ1-4 or with the same dose of an AAV8-GFP as control and sacrificed at 24 weeks after injection. Mortality in *Atp7b^–/–^* mice injected with AAV8-GFP control vector was approximately 50%, whereas the survival rate of those treated with AAV8-Alb-ATP7BΔ1-4 was similar to that of *Atp7b^+/–^* healthy control mice (Figure 1A). Concentrations of serum ALB-2A fusion protein were measured every 4 weeks after dosing and increased in mice injected with AAV8-Alb-ATP7BΔ1-4, suggesting progressive expansion of edited hepatocytes until they reached a plateau of an average of 73.1 μg/mL ALB-2A at week 12 that was maintained throughout the remainder of the study (Figure 1B). Total albumin levels were steady in all mice in treatment and control groups (Supplemental Figure 2A).

At sacrifice, in *Atp7b^–/–^* animals injected with control vector, macroscopic architecture of the livers was aberrant, with several nodular structures. Conversely, livers from AAV8-Alb-ATP7BΔ1-4–injected mice had regular morphology with no gross alterations (Figure 1C). In these mice, liver expression of ATP7BΔ1-4 was confirmed at the protein level (Supplemental Figure 2B) and IHC showed extensive repopulation of the liver by hepatocytes expressing ATP7BΔ1-4 (Figure 1D), resulting in the rescue of serum ceruloplasmin oxidase activity (Figure 1E).

**Figure 2:**
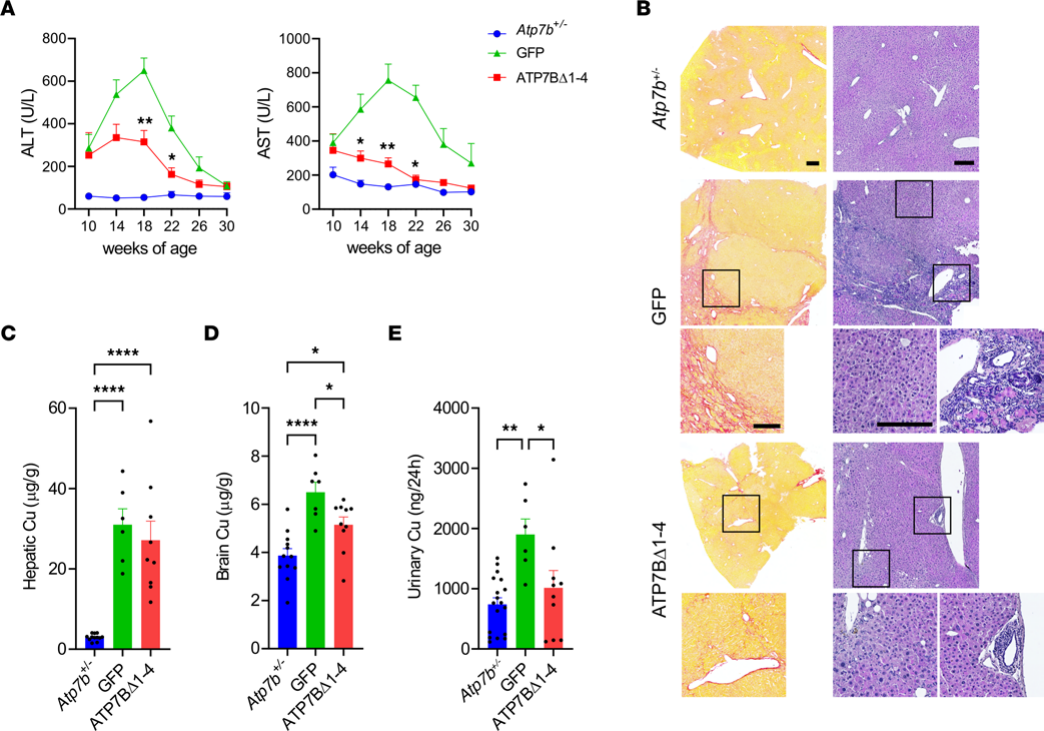
Promoterless mini-ATP7B improves liver pathology and copper metabolism. *Atp7b^–/–^* mice (6 weeks old) were injected with 2.3 × 10^13^ gc/kg AAV8-GFP (GFP) or AAV8-Alb-ATP7BΔ1-4 (ATP7BΔ1-4). *Atp7b^+/–^* mice are shown as heathy controls. (**A**) Serum transaminases levels. Data from animals that survived until the end of the study are shown. For 2-way ANOVA plus Tukey’s post hoc test, *P < 0.05, **P < 0.01 vs. GFP. (**B**) Representative images from liver sirius red (left panels) and H&E (right panels) staining. Scale bars: 200 μm. Analysis of copper content by ICP-MS in (**C**) liver (**D**) brain, and (**E**) urine. For 1-way ANOVA plus Tukey’s post hoc test, *P < 0.05; **P < 0.01; ****P < 0.001.

To investigate the effect of genome editing on copper-induced liver damage, we analyzed circulating levels of transaminases. *Atp7b^–/–^* mice injected with AAV8-GFP showed a transient increase in alanine transaminase (ALT) and aspartate transaminase (AST) levels, peaking at 16 weeks of age, that were significantly reduced in mice injected with AAV8-Alb-ATP7BΔ1-4 (Figure 2A). Peak ALT levels were similar in AAV8-GFP–treated and untreated *Atp7b^–/–^* mice, suggesting AAV8-GFP injection did not result in additional liver damage (Supplemental Figure 2C). Liver pathology revealed large regenerative areas, characterized by normal liver parenchyma, surrounded by areas of intensely inflamed and fibrotic tissue and biliary proliferation in AAV8-GFP–injected mice (Figure 2B). Conversely, livers from AAV8-Alb-ATP7BΔ1-4 injected mice showed mild to moderate inflammation and fibrosis without biliary proliferation (Figure 2B), albeit hepatic copper content was similar to that in AAV8-GFP–injected mice (Figure 2C). Nevertheless, genome editing significantly reduced brain and urinary copper levels (Figure 2, D and E).

### Earlier, promoterless mini-ATP7B administration improved liver repopulation and therapeutic efficacy.

**Figure 3:**
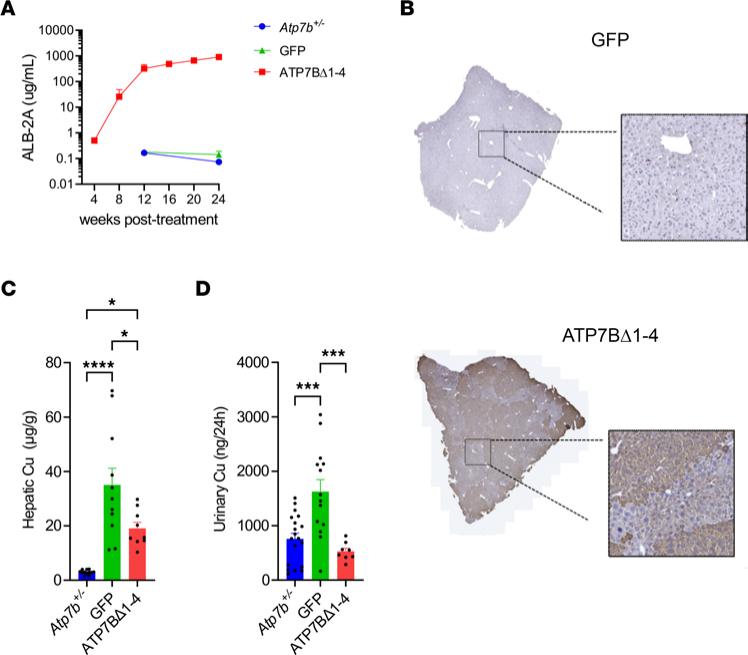
Earlier administration of promoterless mini-ATP7B improves liver repopulation and copper metabolism. *Atp7b^–/–^* mice (2 weeks old) were injected with 2.3 × 10^13^ gc/kg AAV8-GFP (GFP; *n* = 18) or AAV8-Alb-ATP7BΔ1-4 (ATP7BΔ1-4; *n* = 10). *Atp7b^+/–^* mice (*n* = 13) are shown as heathy controls. (**A**) Serum levels of ALB-2A fusion protein. (**B**) Representative images from liver IHC analysis using anti–ATP7B Ab. Analysis of copper content by ICP-MS in (**C**) liver and (**D**) urine. For 1-way ANOVA plus Tukey’s post hoc test, *P < 0.05; ***P < 0.005; ****P < 0.001.

To further improve the therapeutic efficacy, we treated *Atp7b^–/–^* mice at 2 weeks of age with AAV8-Alb-ATP7BΔ1-4 vector to exploit physiological liver growth in addition to the selective growth advantage of corrected hepatocytes. Consistent with previous experiments in juvenile mice, genome editing rescued survival in *Atp7b^–/–^* mice (Supplemental Figure 3A) and increased circulating ALB-2A levels (Figure 3A). When compared with mice dosed at 6 weeks of age, mice dosed at 2 weeks of age with AAV8-Alb-ATP7BΔ1-4 had 1-log higher ALB-2A levels at the end of the study (924.2 μg/mL ALB-2A for mice dosed at 2 weeks compared with 80.6 μg/mL ALB-2A in mice dosed at 6 weeks). Kinetics of the increase in ALB-2A were comparable between the groups (Figure 3A), and total albumin levels, again, were consistent over time for the animals in both the treatment and control groups (Supplemental Figure 3B). At 24 weeks after treatment, livers had normal macroscopic morphology (data not shown) and IHC showed extensive liver repopulation by ATP7BΔ1-4–expressing hepatocytes, which was larger than in 6-week-old injected animals (Figure 3B), consistent with increased ALB-2A levels. Treatment with AAV8-Alb-ATP7BΔ1-4 reduced transaminase activities in *Atp7b^–/–^* mice (Supplemental Figure 3C). Liver pathology showed areas of advanced fibrosis and intense inflammation, together with liver regeneration and biliary proliferation in *Atp7b^–/–^* mice treated with the control vector, whereas moderate liver fibrosis and inflammation were observed in mice injected with AAV8-Alb-ATP7BΔ1-4 (Supplemental Figure 3D) that also showed a concomitant significant decrease in hepatic and urinary copper levels compared with AAV8-GFP–treated livers (Figure 3, C and D). *Atp7b^–/–^* mice did not show copper accumulation in the brain at this stage (Supplemental Figure 3E).

### Promoterless mini-ATP7B ameliorated liver pathology and copper storage in a milder WD mouse model.

**Figure 4:**
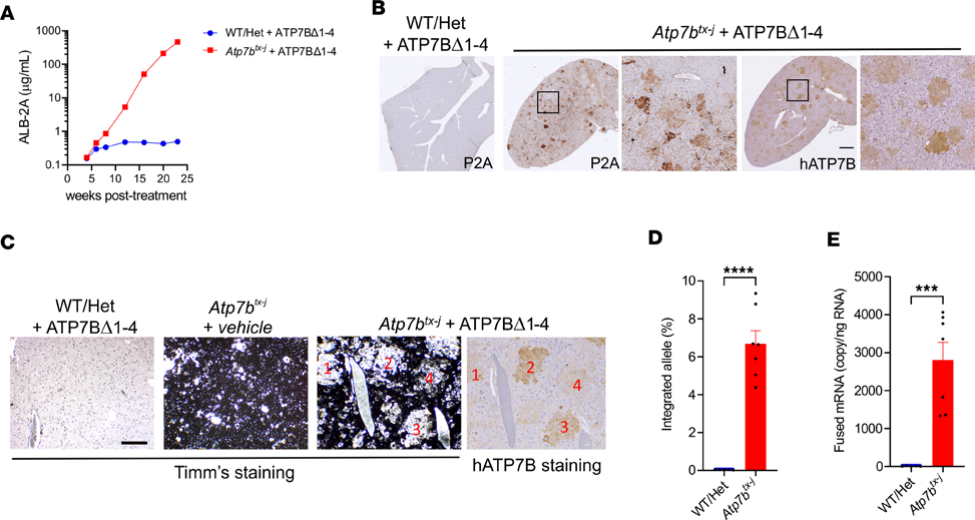
Promoterless mini-ATP7B confers selective advantage to *Atp7b^tx-J^* mouse hepatocytes. *Atp7b^tx-J^* and WT or heterozygous (Het) healthy control mice (3 weeks old) were injected with 1 × 10^14^ vg/kg AAV-DJ-Alb-ATP7BΔ1-4 (ATP7BΔ1-4) or vehicle (*n* = 5–7/group). Tissues were harvested when mice were 25 weeks old. (**A**) Expression of ALB-2A levels in serum. (**B**) Representative images of IHC liver staining for P2A and human ATP7B. Scale bar: 1 mm. (**C**) Timm’s and human ATP7B IHC staining on consecutive liver slices. Numbers denote corresponding areas. Scale bar: 250 μm. (**D**) On-target genomic DNA integration analysis. (**E**) Fusion mRNA analysis. For 2-tailed *t* test, ***P < 0.005, ****P < 0.001.

To further explore the therapeutic potential of genome editing for the treatment of WD, we investigated AAV-mediated delivery of promoterless ATP7BΔ1-4 in the toxic-milk mouse from The Jackson Laboratory (*Atp7b^tx-J^*), which carries a spontaneous missense pathogenic variant in *Atp7b* resulting in a glycine-to-aspartate change at amino acid position 712 (11, 12). This model displays a milder phenotype, characterized by slower disease progression compared with the full KO model; the survival rate of *Atp7b^tx-J^* mice is not altered after 1 year of monitoring (Supplemental Figure 4A). *Atp7b^tx-J^* and healthy control mice were injected with 1 × 10^14^ gc/kg AAV-DJ-Alb-ATP7BΔ1-4 or vehicle at 3 weeks of age and sacrificed 22 weeks later. Consistent with findings from *Atp7b^–/–^* mice, treatment with the AAV-DJ-Alb-ATP7BΔ1-4 vector resulted in a progressive increase of ALB-2A fusion protein in serum (Figure 4A), whereas total serum albumin levels remained steady (Supplemental Figure 4B). IHC analysis also showed repopulation of the liver by hepatocytes expressing the fused P2A tag and human mini-ATP7B (Figure 4B), and serum ALB-2A levels correlated with the ratio of edited hepatocytes (Supplemental Figure 4, C and D). Moreover, Timm’s and ATP7B histochemical staining conducted in consecutive liver sections showed extensive and homogeneous copper accumulation in untreated mice, whereas sections from AAV-DJ-Alb-ATP7BΔ1-4–treated mice had clustered cells expressing human ATP7B and were devoid of copper staining (Figure 4C). Analysis of targeted alleles at the genomic DNA level (Figure 4D) and fusion mRNA (Figure 4E) showed detectable integration of AAV-DJ-Alb-ATP7BΔ1-4 at the *Alb* locus.

**Figure 5:**
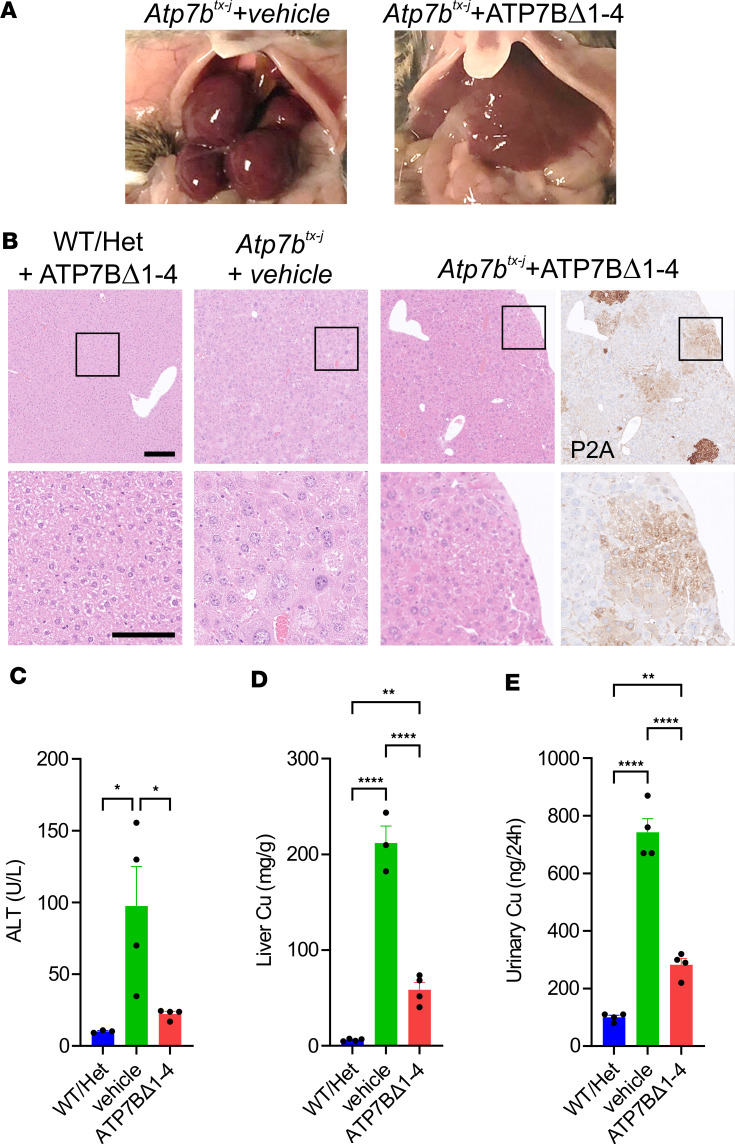
Genome editing with promoterless mini-ATP7B reduces copper levels and improves liver disease in *Atp7b^tx-J^* mice. *Atp7b^tx-J^* mice (4 weeks old) and WT or heterozygous (Het) healthy control mice were injected with 1 × 10^14^ vg/kg AAV-DJ-Alb-ATP7BΔ1-4 (ATP7BΔ1-4) or vehicle and sacrificed at the age of 42 weeks (*n* = 4 per group). (**A**) Representative photographs of livers at sacrifice. (**B**) Representative images from H&E and human ATP7B histochemical staining. Scale bars: 200 μm and 100 μm (high-magnification insets). (**C**) Serum ALT levels. Copper content by ICP-MS in (**D**) liver and (**E**) urine. For 1-way ANOVA plus Tukey’s post hoc test, *P < 0.05; **P < 0.01; ****P < 0.001.

To investigate the effect of genome editing on liver disease and copper storage, 4-week-old mice were injected with 1 × 10^14^ gc/kg AAV-DJ-Alb-ATP7BΔ1-4 and sacrificed at 36 weeks of age. In contrast to the nodular appearance of untreated *Atp7b^tx-J^* mouse livers, livers from genome-edited mice had normal macroscopic morphology and cell size (Figure 5, A and B). Furthermore, treatment with AAV-DJ-Alb-ATP7BΔ1-4 significantly improved liver damage, with repopulated areas expressing human mini-ATP7B showing normal cell morphology (Figure 5B), normalized serum ALT levels (Figure 5C), and reduced copper concentrations in urine and liver (Figure 5, D and E).

To track the progression of liver repopulation on a molecular level, mice were dosed 4 weeks after birth with either vehicle or 1 × 10^14^ vector genomes (vg)/kg AAV-DJ-Alb-ATP7BΔ1-4. Terminal samples from a subset of mice in each group were taken 8, 16, and 24 weeks after dosing. Assessment of vector genome copy per cell and percentage of integrated cells was performed. No signal for either metric was noted in vehicle-treated animals, but decreasing vector genome copies and increasing percentage of integrated cells over time were noted in treated animals (Supplemental Figure 5, A and B). Additionally, quantification of the percentage of corrected hepatocytes expressing P2A in animals sacrificed at ages 25 and 42 weeks clearly indicates expansion of corrected hepatocytes over time (Supplemental Figure 5, C and D). Taken together, these data suggest that promoterless ATP7BΔ1-4–based genome editing resulted in liver repopulation by edited hepatocytes and disease correction in a milder WD model.

### Promoterless mini-ATP7B in combination with chelation therapy corrected WD.

**Figure 6:**
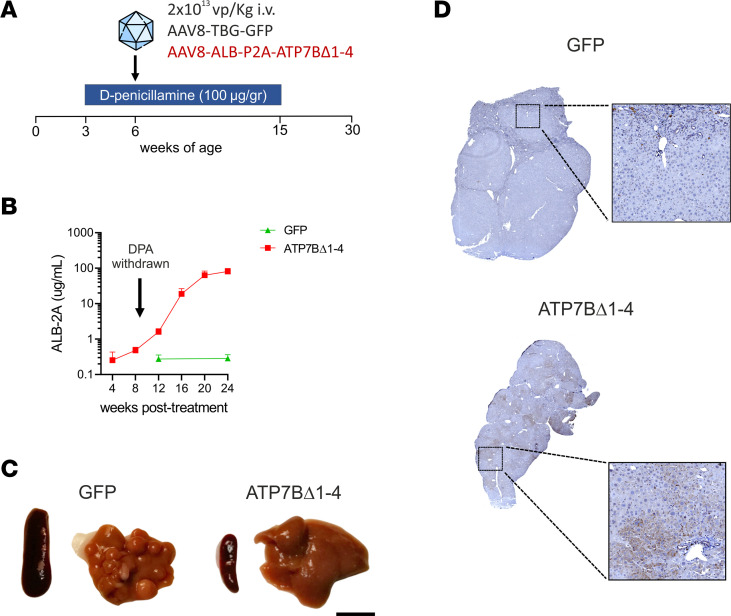
Integration of promoterless mini-*ATP7B* confers proliferative advantage in WD mice under chelator therapy. *Atp7b^–/–^* mice (6 weeks old) were treated with DPA and then injected with 2.3 × 10^13^ gc/kg of AAV8-GFP (GFP; *n* = 6) or AAV8-Alb-ATP7BΔ1-4 (ATP7BΔ1-4; *n* = 6). *Atp7b^+/-^* mice (*n* = 13) are shown as heathy controls. (**A**) Experimental timeline. (**B**) Serum levels of ALB-2A fusion protein. (**C**) Representative photographs of dissected livers and spleens. Scale bar: 1 cm. (**D**) Representative images from liver immunohistochemistry using anti-ATP7B Ab.

To mimic the clinical conditions, considering that most of the patients who could be enrolled in a gene therapy clinical trial are receiving standard chelation therapy, we next investigated genome editing in mice treated with the currently available therapy for WD. For this aim, we administered mice with d-penicillamine (DPA) in drinking water (13). Starting from weaning, *Atp7b^–/–^* mice were administered 100 μg/g BW DPA and then they were injected with 2.3 × 10^13^ gc/kg AAV8-Alb-ATP7BΔ1-4 or AAV8-GFP at age 6 weeks. DPA was withdrawn 9 weeks later, and animals were sacrificed 24 weeks after vector administration (Figure 6A).

Survival rates were similar among experimental groups (Supplemental Figure 6A). ALB-2A levels in AAV8-Alb-ATP7BΔ1-4–treated mice increased to reach an average concentration of 82.5 μg/mL at 24 weeks after dosing. Although the final value of ALB-2A reached with DPA was similar to that in mice without DPA, the kinetics of ALB-2A increase were different, with expansion of edited hepatocytes (as seen by increasing ALB-2A levels) occurring largely after DPA removal, 12 weeks after dosing (Figure 6B), whereas log increase of ALB-2A was already detected by 4–8 weeks after vector administration in the groups without DPA (Figure 1B and Figure 3B). This finding is consistent with selective pressure driving repopulation by edited cells. Total albumin levels were again steady throughout the study in all animals treated with DPA (Supplemental Figure 6B).

**Figure 7:**
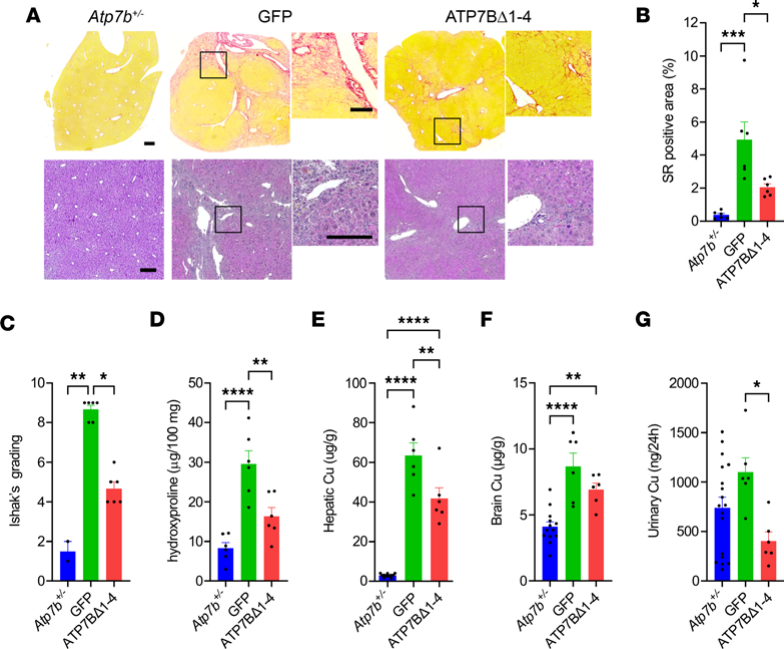
Combination of genome editing and chelator therapy ameliorates WD. *Atp7b^–/–^* mice (6 weeks old) were treated with DPA and then injected with 2.3 × 10^13^ gc/kg of AAV8-GFP (GFP; n = 6) or AAV8-Alb-ATP7BΔ1-4 (ATP7BΔ1-4; *n* = 6). *Atp7b^+/–^* mice (*n* = 13) are shown as heathy controls. (**A**) Representative images from liver sirius red (upper panels) and H&E (lower panels) staining. Scale bars: 200 μm. (**B**) Quantitative morphometry of sirius red (SR) staining. (**C**) Ishak’s necroinflammatory score. (**D**) Hydroxyproline (HYP) content analysis. Copper content analysis by ICP-MS in (**E**) liver, (**F**) brain, and (**G**) urine. One-way ANOVA plus Tukey’s post hoc test (**B** and **D**–**F**) or Kruskal-Wallis test plus Dunn’s post hoc test (**C**). *P < 0.05; **P < 0.01; ***P < 0.005; ****P < 0.001.

At sacrifice, animals treated with AAV8-GFP had compromised liver structure and splenomegaly, whereas liver and spleen of AAV8-Alb-ATP7BΔ1-4–injected mice had regular morphology and size (Figure 6C) and areas of repopulation by ATP7BΔ1-4–expressing hepatocytes (Figure 6D). After DPA withdrawal, circulating transaminase levels further increased in *Atp7b^–/–^* mice injected with AAV8-GFP, whereas they remained stable in animals injected with the editing vector (Supplemental Figure 6C). Moreover, genome editing resulted in significant amelioration of liver pathology (Figure 7, A–D) and copper metabolism (Figure 7, E–G).

To study the correlation between liver damage and repopulation by edited hepatocytes in *Atp7b^–/–^* animals treated with AAV8-Alb-ATP7BΔ1-4, we analyzed serum ALT and ALB-2A levels as markers of liver injury and edited hepatocyte expansion, respectively. *Atp7b^–/–^* mice were fed a low-copper diet to alleviate the toxic burden of copper in tissues and to further modulate WD progression. Maximum ALT levels were similar among experimental groups, although ALT levels peaked later in animals fed the low-copper diet or treated with DPA compared with mice fed with a high-copper diet and not treated with DPA (Supplemental Figure 7A). Consistently, ALB-2A achieved similar levels in all animals dosed with the genome-editing vector at age 6 weeks, but DPA and a low-copper diet resulted in delayed ALB-2A increase (Supplemental Figure 7B). Moreover, comparison of ALT and ALB-2A kinetics revealed that the fastest ALB-2A increases occurred after the ALT peak and reached a plateau when ALT levels declined (Supplemental Figure 7, C–E). These findings suggest that the severity of liver damage modulates repopulation by ATP7BΔ1-4–edited hepatocytes in *Atp7b^–/–^* mice. Nevertheless, in contrast to mice dosed at 6 weeks of age, animals treated at age 2 weeks had ALB-2A levels that steadily increased over time, irrespective of changing ALT levels (Supplemental Figure 7F), and ALT kinetics was similar to animals injected at 6 weeks of age (Supplemental Figure 7A).

## Discussion

In the absence of treatment, WD is a progressive and fatal disorder. Thankfully, since the 1950s, patients with WD have benefited from medical therapy to arrest disease progression or prevent complications, and copper chelators and zinc salts have saved thousands of lives, ensuring patients with WD a decent quality of life. Nevertheless, these life-saving treatments have several limitations that became evident over the years, with up to 50% of patients having periods of nonadherence because of side effects and liver failure or neuropsychiatric symptom worsening in a subset of patients. Liver transplantation is curative for WD, and this has laid the basis for the development of liver-directed gene therapy for the treatment of WD. Here, we provided proof of concept for liver-directed, nuclease-free genome editing with a promoterless donor DNA correcting WD. This strategy has been successfully applied to few other diseases, including hemophilia B (8), Crigler-Najjar syndrome type I (14), and methylmalonic acidemia (15). This technology exploits the strong and liver-specific *Alb* promoter to drive transgene expression coupled with the ability of corrected hepatocytes to repopulate the liver, if editing results in a growth advantage over noncorrected hepatocytes.

Early hepatocyte transplantation studies in WD rodent models suggested the possibility that ATP7B-expressing hepatocytes could repopulate ATP7B-deficient liver and correct WD. However, these experiments included treatments that induced hepatocyte proliferation (e.g., partial hepatectomy) and administration of toxins to reduce regrowth of the native cells (16). Here, we demonstrated that corrected hepatocytes hold a strong proliferative advantage in the absence of preconditioning treatments in WD livers. Repopulation was achieved in both *Atp7b^–/–^* and *Atp7b^tx-J^* mice, but at different rates, supporting the hypothesis that severity of liver damage may determine repopulation speed and extent. This was further confirmed by combining genome editing with copper chelator therapy, which resulted in attenuated liver damage, a slower rate of increase of ALB-2A, and less extensive liver repopulation being observed under this condition compared with genome editing alone. Moreover, *Atp7b^–/–^* mice fed a low-copper diet had a slower rate of increase of ALB-2A compared with mice fed a high-copper diet, thus providing further evidence that the rate of expansion of genome-edited cells depends on the extent of liver damage. Importantly, repopulation also seems to occur during chelation therapy. While supporting the feasibility of genome-editing vector administration in patients under chelation therapy, this evidence also paves the way for future studies aimed at assessing appropriate chelator-tapering protocols to enhance repopulation rate while controlling copper-mediated liver damage.

Dosing mice with AAV-Alb-ATP7BΔ1-4 at younger ages increased the final ALB-2A plateau level in both the *Atp7b^–/–^* and *Atp7b^tx-J^* models (data not shown). Moreover, early administration of AAV-Alb-ATP7BΔ1-4 seems to result in damage-independent liver repopulation, although the limitation of using serum ALT levels as a liver damage index should be considered. Several mechanisms may contribute to this differential modulation of the expansion of edited hepatocytes depending on dosing age. First, HDR is considered more likely to occur in actively dividing cells (17), which could be expected in younger mice. However, this assumption has been recently challenged by in vivo findings showing spontaneous HDR occurring at higher frequency in hepatocytes that did not progress through S phase (18). However, it should be noted that this study was performed in WT animals at 4 weeks of age, with most of the hepatocytes in G_0_ (18), which represents a quite different context compared with livers at earlier postnatal stages or affected by WD, when hepatocyte proliferation is expected to be more extensive (19). Second is hepatocyte polyploidization during early postnatal development (20, 21), which could result in the multiplication of targeted alleles. Third, different populations of hepatocytes with different duplication capacities were targeted depending on the age of administration. And fourth is reduced AAV transduction in older and more damaged livers. Edited hepatocytes in younger mice may also have an increased proliferative advantage over edited hepatocytes in older mice, given the natural growth of the liver as young mice age. Whatever the cause, this difference highlights the importance of early dosing to achieve better therapeutic outcomes in WD.

In our study, the vector dose used for *Atp7b^–/–^* mice was within a clinically relevant range, whereas a higher vector dose was required to achieve disease correction in *Atp7b^tx-J^* mice with a milder phenotype and slower repopulation rate. However, several strategies could be applied to increase the potency of the proposed treatment and make it more attractive for clinical translation, including transient administration of pharmacological HDR enhancers to increase the integration rate (18, 22), or switching to a vector capsid with higher transduction efficiency to human hepatocytes (23).

Despite the extensive liver repopulation observed in *Atp7b^–/–^* mice injected at 6 weeks of age, we did not observe reduction of hepatic copper compared with untreated animals. This is not surprising, because copper does not continuously accumulate in *Atp7b^–/–^* mouse liver; it progressively declines starting from 2 months of age (19). Moreover, copper storage in the *Atp7b^–/–^* mouse liver is uneven, with regenerative areas showing negligible copper accumulation (24) that redistributes from hepatocytes to nonparenchymal cells (25). Nevertheless, we showed that genome editing effectively reduced hepatic copper deposits after earlier dosing in *Atp7b^–/–^* and in *Atp7b^tx-J^* mice.

AAV inoculation in newborn mice has been associated with the development of hepatocellular carcinoma (HCC) due to random AAV integration, with a mechanism involving transactivation by vector-borne promoter elements of multiple miRNAs embedded within the mouse *Rian* locus (26). Moreover, preexisting liver injury appears to increase the incidence of HCC after AAV gene therapy in adult mice (27). Even though no evidence of HCC has been reported in either human or nonhuman primate studies involving AAV vectors (28), this may be due to the relatively few individuals investigated and short time of observation after treatment. Gene transfer to injured livers has never been investigated in large animal models or in humans and, in addition to liver injury, WD is also characterized by copper-mediated DNA damage (29–31), which could increase undesired random AAV integration events within the hepatocyte genome. Therefore, patients with WD may be at higher risk to develop HCC as a consequence of gene therapy when episomal AAV vectors are used. In contrast, promoterless AAV vectors are expected to have a more favorable safety profile because they would not transactivate gene-flanking integration sites, although analysis of off-target integration events and tumorigenesis studies should be performed to further assess their safety. Although the growing body of data from human patients dosed with AAV will progressively define genotoxic risks associated with AAV-based therapies, promoterless, nuclease-free genome editing may represent an effective and potentially safer alternative to classic gene therapy, especially for pediatric patients. In line with safety, our findings also confirmed that targeted integration at the *Alb* locus of promoterless AAV does not perturb albumin production.

WD is characterized by a very broad spectrum of clinical manifestations and disease progression rates. Moreover, most patients benefit from currently available therapies that modify the natural history of the disease. In this work, to address this extensive clinical variability and evaluate the translational potential of our approach, we used 2 mouse models characterized by different disease severity and progression and evaluated genome editing in combination with chelation therapy. However, some aspects of the disease have not been investigated here and must be considered in the progression toward the clinic. One aspect is related to liver fibrosis, which is present at diagnosis in almost all patients with WD and may hamper AAV-mediated gene transfer, thus reducing therapeutic efficacy. Second, although they mimic most of the hepatic phenotypes observed in humans, WD mice (and any currently available WD model) lack an overt neurological phenotype, thus preventing the investigation of therapeutic efficacy on this relevant aspect of WD. Future studies successfully addressing these 2 issues will further expand the potential target population for genome editing and, generally, for gene therapy. In conclusion, promoterless, nuclease-free genome editing with mini-ATP7B provided a significant and sustained therapeutic benefit in WD and may represent a safer and longer-lasting alternative to classic gene replacement strategies.

## Methods

### AAV vectors.

DNA constructs bearing sequences encoding codon-optimized human ATP7BΔGln70-Cys499, flanked by 5′ (chr5: 90,622,517–90,623,166) and 3′ (chr5: 90,623,170–90,623,819) *Alb* HAs and preceded by the P2A-encoding sequence (32), were synthesized (Genewiz) and cloned into a pAAV2.1 plasmid. Serotype 8 AAV vector was produced by triple transfection of HEK293 cells, as previously described (33) (InnovaVector), and administered to *Atp7b^–/–^* mice. AAV-TBG-GFP vector was used as a control for the injection.

For experiments in *Atp7b^tx-J^* mice, a slightly different vector was used. Specifically, this vector carried the human *ATP7B* DNA sequence with a similar truncation region (ΔVal57-Ala486) and similar *Alb*-flanked sequences as HAs except the flanked sequences are 50 bp shorter at both the 5′ and 3′ ends. The AAV-DJ (34) vector was produced similarly by triple transfection of HEK293F cells (Thermo Fisher Scientific).

### Mouse studies.

*Atp7b^–/–^* (35) mice were housed at the Telethon Institute of Genetics and Medicine (TIGEM) animal facility and received food and water ad libitum. Mice were fed a VRFI (P) standard diet (Special Diet Services) or the 1324 Maintenance diet (Altnormin) as the low-copper diet. Chow copper content was 22–34 mg/kg and 13 mg/kg, respectively. Only male mice were used for experimental procedures to reduce experimental variability derived from sex-associated differences in WD severity and in hepatocyte transduction efficiency by AAV. AAV vectors were administered by i.v. injection in 0.9% NaCl solution to male mice at 2 and 6 weeks of age. For urine collection, mice were placed in metabolic cages for 24 hours. Insoluble or suspension particles were removed by centrifugation, and complete urine collection was confirmed by creatinine levels. At sacrifice, animals were perfused with PBS and livers were harvested for further analysis. Serum ceruloplasmin activity was investigated as previously described (36). *Atp7b^tx-J^* mice carrying the point mutation G712D were purchased from The Jackson Laboratory (catalog 001576) and housed at the LogicBio animal facility in accordance with the LogicBio IACUC. Mice received food and water ad libitum. AAV was similarly administered i.v. in solutions consisting of 150 NaCl, 10 μM Na_3_PO_4_, and 0.001% Pluronic F68. Serum and urine samples were collected every 2 weeks.

### Western blot.

Liver samples were lysed in RIPA buffer and incubated for 30 minutes on ice, supplemented with protease inhibitor cocktail (Sigma-Aldrich) and centrifuged for 20 minutes. Supernatant was collected and protein content was determined by Bradford assay (Bio-Rad). Protein samples were separated by SDS-PAGE using 4%–12% polyacrylamide gels (Bio-Rad). Primary Ab rabbit anti-ATP7B (Abcam, catalog ab124973; dilution 1:100) and mouse anti-GAPDH (Santa Cruz Biotechnology, catalog sc-32233; dilution 1:1000) were diluted in TBS-T (0.8% NaCl, 0.02% KCl, 0.3% Tris-base, 0.1% Tween 20) in 5% milk (Bio-Rad). Proteins of interest were detected with HRP-conjugated goat anti–mouse IgG Ab (GE Healthcare). Peroxidase substrate was provided in the ECL Western Blotting Substrate Kit (Pierce).

### Hydroxyproline content analysis.

Hepatic hydroxyproline content was measured as previously described (37). Briefly, homogenized liver tissue was hydrolyzed in 6N HCl at 110°C for 16 hours. Hydrolysates were filtered and assayed in citrate-acetate buffer. Samples were incubated with chloramine-T solution (Sigma-Aldrich) for 20 minutes at room temperature (RT). Next, Ehrlich’s reagent (Sigma-Aldrich) was added, samples were incubated at 65°C for 20 minutes, and absorbance was measured at 550 nm.

### Liver staining.

Livers from PBS-perfused mice were fixed in 4% paraformaldehyde for 12 hours, stored in 70% ethanol, and embedded in a paraffin block. H&E staining was performed on 5 μm–thick paraffin sections of livers, which were rehydrated and stained in Mayer’s hematoxylin (Bio-Optica) for 4 minutes. After 2 washes in tap water for 5 minutes, sections were incubated in a solution of 0.1% ammonia water (1 mL of ammonium hydroxide in 1 L of distilled water) for 1 minute, washed again in tap water for 5 minutes, and counterstained in eosin Y solution (Sigma-Aldrich) for 30 seconds. Sections were dehydrated, cleared in xylene, and mounted in a resinous medium. Images were captured by Axio Scan.Z1 microscope (Zeiss). Sections were evaluated by a blinded experienced pathologist for necroinflammatory grading using Ishak’s scoring system (38).

For sirius red staining, 5 μm–thick sections were rehydrated and stained for 1 hour in picrosirius red solution (0.1% sirius red in saturated aqueous solution of picric acid). After 2 changes of acidified water (0.5% acetic acid in water), sections were dehydrated, cleared in xylene, and mounted in a resinous medium. Images were captured by Axio Scan.Z1 microscope and analyzed using ImageJ software (NIH) for quantification of sirius red–positive areas. Five images for each mouse were analyzed.

For IHC, 5 μm–thick sections were rehydrated and permeabilized in PBS/0.2% Triton X-100 (Sigma-Aldrich) for 20 minutes. Antigen unmasking was performed in 0.01 M citrate buffer in a microwave oven. Next, sections underwent blocking of endogenous peroxidase activity in methanol/1.5% H_2_O_2_ (Sigma-Aldrich) for 30 minutes and incubated with blocking solution (3% BSA, Sigma-Aldrich; 5% donkey serum, Millipore; 1.5% horse serum, Vector Laboratories; 20 mM MgCl_2_; and 0.3% Triton X-100 [Sigma-Aldrich] in PBS) for 1 hour. Sections were incubated with primary Ab (rabbit anti-ATP7B, 1:100 dilution; Thermo-Fisher Scientific, catalog PA-102826) overnight at 4°C and then with universal biotinylated horse anti–mouse/rabbit IgG secondary Ab (Vector Laboratories) for 1 hour. Biotin/avidin-HRP signal amplification was achieved using the ABC Elite Kit (Vector Laboratories) according to manufacturer’s instructions. DAB (Vector Laboratories) was used as the peroxidase substrate. Mayer’s hematoxylin (Bio-Optica) was used for counterstaining. Sections were dehydrated and mounted in Vectashield (Vector Laboratories). Image capture was performed using an Axio Scan.Z1 microscope.

For IHC of albumin-2A, an anti–P2A Ab was custom raised and used at 1:100 dilution. In Figure 4, the anti-human ATP7B Ab used was purchased from Abcam (catalog ab124973; 1:100 dilution). Slides were incubated with primary Ab for 1 hour at RT, followed by incubation with Ultra Streptavidin HRP Kit according to manufacturer’s instruction (BioLegend). Slides were imaged using a digital slide scanner (Hamamatsu) or a compound microscope (AmScope). Images were quantified using ImageJ.

### Timm’s copper staining.

Slides were deparaffinized with xylene and rehydrated with ethanol and water. Slides were then incubated with 0.5% ammonium sulfide (VWR) for 5 minutes at RT, rinsed with water, and incubated with 0.1N HCl for 3 minutes. Slides were incubated with the developer solution for 10 minutes. The developer solution was made of 1 part 5% silver nitrate (VWR) and 5 parts of a solution consisting of 2% wt/vol hydroquinone (Thermo Fisher Scientific) and 5% wt/vol citric acid (Thermo Fisher Scientific).

### Copper analysis.

Copper content analysis was performed by inductively coupled plasma mass spectrometry (ICP-MS) on wet brain and liver tissue and urine samples. Samples were mineralized as follows: concentrated nitric acid and hydrochloric acid (Romil) were added to each sample at a 1:3 ratio and then incubated for 16 hours at 90°C. Samples were diluted to 15 mL in milli-Q water and transferred into ICP-MS vials for analysis. Copper analysis was performed in triplicate using a 7700 ICP-MS instrument (Agilent Technologies) equipped with a frequency-matching radiofrequency generator and third-generation Octapole Reaction System, operating with helium gas in ORF. The following parameters were used: radiofrequency power, 1550 W; plasma gas flow, 14 L/min; carrier gas flow, 0.99 L/min; and helium gas flow, 4.5 mL/min. ^103^Rh was used as an internal standard (final concentration, 50 μg/L). Standard solutions of proper metal were prepared in 3% nitric acid at 5 different concentrations (0, 1, 10, 50, and 100 μg/L). For copper content measurement in *Atp7b^tx-J^* mice, the ICP-MS analysis was carried out on wet tissue samples similarly by the Core Research Facilities at the University of Massachusetts Lowell. These procedures were described previously (39).

### Alanine aminotransferase analysis.

Serum transaminase levels were measured by Scil Vitro Vet analyzer (Scil Vet) for studies in *Atp7b^–/–^* mice and Alanine Aminotransferase Activity Colorimetric/Fluorometric Assay Kit (Biovision) for *Atp7b^tx-J^* mice, according to the manufacturer’s instructions.

### Genomic DNA integration assessment.

To assess genomic DNA (gDNA) integration, DNA was extracted from liver-tissue punches using the DNeasy Blood and Tissue kit (Qiagen) according to the manufacturer’s instructions. DNA was quantified using a NanoDrop (NanoDrop One, ND-ONE-W) and all DNA samples were diluted to a final concentration of 50 ng/μL. Long-range PCR was performed on this DNA using a forward primer (5′-ATGTTCCACGAAGAAGCCA-3′) that complements the sequence upstream of the albumin HA included in the AAV vector, and a reverse primer (5′-TCAGCAGGCTGAAGTTGGT-3′) that complements the P2A sequence. Q5 High-Fidelity DNA Polymerase (New England Biolabs) was used for the long-range PCR according to the manufacturer’s specifications. The long-range PCR product was purified using SPRI (Beckman Coulter) beads according to the manufacturer’s specifications for the anticipated product size of the long-range PCR. qPCR was then performed on the purified long-range PCR product using the forward primer (5′-ATGTTCCACGAAGAAGCCA-3′) from the long-range PCR, which sits upstream of the 5′ HA, and a primer and TaqMan-FAM probe within the 5′ end of the HA (reverse primer 5′-AGCTGTTTCTTACTCCATTCTCA-3′; probe: 5′-AGGCAACGTCATGGGTGTGACTTT-3′). qPCR reactions were performed in TaqMan Universal PCR Master Mix (Applied Biosystems) according to the manufacturer’s specifications. qPCR was run on a QuantStudio 5 real-time PCR systems (Applied Biosystems). Final percentage integration was determined by comparing sample values against an 8-point standard curve generated by spiking in gBlocks of the long-range PCR product into WT mouse gDNA. Although gDNA was normalized on the basis of concentration before the long-range PCR, a qPCR reaction against the mouse gene transferrin receptor 1 (*Tfrc*) was performed to allow for a semiquantitative readout of the percentage of cells that were edited. The sequences used for primers and probes for the *Tfrc* normalization reaction are as follows: forward primer (5′-CCTGGTATATGTGGAGGTTTGAG-3′), reverse primer (5′-GGATTTAGAGCAGGAGCCTTAC-3′), and probe (5′-TGGCAGTGAGTTCTTCCCACCATG-3′).

### Integrated mRNA analysis.

Liver mRNA was extracted using TRIzol (Thermo Fisher Scientific) and was further purified using the RNeasy Kit (Qiagen). Transcript from the integrated transgene was quantified by reverse transcription–coupled droplet digital PCR (ddPCR) using a qualified method with a forward primer (F2), a reverse primer (R3), and a probe (P2) using the ddPCR kit (Bio-Rad). The primers and probes for mouse experiments are F2m (5′-CACACTTCCAGAGAAGGAGAAGC-3′), for R3m (5′-TCAGCAGGCTGAAGTTGGT-3′), and P2m (5′-AAGACGCCTTAGCCGGCAGCGGC-3′).

### Determination of vector genome copy number.

To assess vector genome copy number, DNA was extracted from liver-tissue punches using the DNeasy Blood and Tissue Kit (Qiagen) according to the manufacturer’s instructions. DNA was quantified using a NanoDrop One and all DNA samples were diluted to a final concentration of 0.5 ng/μL. ddPCR reactions were prepared with ddPCR Supermix for Probes (no dUTP) (Bio-Rad) according to the manufacturer’s instructions. Primer and probe sets targeting the 5′ HA of the vector and transferrin receptor 1 (*Tfrc*), which served as the control to account for cell number, were used. The sequences used for primers and probes for the reaction targeting the 5′ HA are as follows: forward primer (5′-GATGTCAGAGAGCCTGCTTTAG-3′), reverse primer (5′-AGGACTGTTAGGATTTGCACTT-3′), and probe (5′-AAGCAATGCAAGGCACGTACGTTT-3′). The sequences used for primers and probes for the *Tfrc* normalization reaction are as follows: forward primer (5′-CCTGGTATATGTGGAGGTTTGAG-3′), reverse primer (5′-GGATTTAGAGCAGGAGCCTTAC-3′), and probe (5′-TGGCAGTGAGTTCTTCCCACCATG-3′). Once ddPCR reactions were assembled, droplets were generated in an automated droplet generator (Bio-Rad, catalog 1864101).

Within 1 hour of droplet generation completion, plates were placed in a C1000 thermocycler (Bio-Rad) for PCR. Conditions used for the PCR reaction are those recommended for use with the ddPCR Supermix for Probes (no dUTP) master mix. After completion of the PCR reaction, droplets were read on a QX200 droplet reader (Bio-Rad). Once absolute copy numbers of *Tfrc* and the HA were obtained, copy numbers for *Tfrc* were divided by 2 to generate an estimate of the number of cells (note that diploidy is assumed). Once the approximate cell number was known, copies detected for the HA were divided by the number of cells to determine the vector genomes per cell.

### Plasma ALB-2A measurements.

Plasma ALB-2A levels were measured using a sandwich ELISA. Briefly, plates were coated with 50 μL of a rabbit monoclonal Ab (final concentration, 3 μg/mL in 1× PBS) raised against P2A peptide for 16–18 hours at 4°C. Plates were blocked with 300 μL of 1% milk in PBS per well for 1.5 hours at 37°C. Plasma samples were analyzed at a dilution of 1:100 (for heterozygous untreated and WD mice treated with an AAV-GFP control vector) and 1:1000 (for later time points of WD mice treated with an AAV bearing a mini-ATP7B). Plasma samples were diluted into 1% BSA in 1× PBS plus Tween (PBST). After blocking, plates were washed 4 times with 300 μL of 1× PBST using a microplate washer (BioTek 405 TS), and 50 μL of an 8-point standard or samples were added to appropriate wells in the assay plates. Samples were incubated for 1.5–2 hours at RT on a microplate shaker set to 400 rpm. After sample binding was completed, plates were washed 4 times with 300 μL of 1× PBST using the plate washer. An HRP-conjugated anti–mouse albumin Ab (Abcam, ab19195) was used as the detection Ab and diluted to a concentration of 0.05 μg/mL in 0.1% milk in 1× PBST. Detection Ab (50 μL) was added to the plate and the plate was covered with a foil seal. The detection Ab was incubated for 1.5 hours at RT on a microplate shaker set to 400 rpm. Once incubation with the detection Ab was completed, the plate was washed 4 times with 300 μL of 1× PBST. SuperSignal ELISA Pico Chemiluminescent Substrate (Thermo Fisher Scientific) was used as a developing reagent and enhancer and peroxide reagents were mixed 1:1 per manufacturer’s instructions. Developing reagent (100 μL) was added and incubated on the plate for 3–5 minutes. Luminescence values were read on a plate reader (Clariostar Plus).

### Mouse plasma total albumin levels.

Mouse plasma total albumin levels were measured using a sandwich ELISA. Plates were coated with an anti–mouse albumin Ab (Abcam, catalog ab19194). The anti–mouse albumin Ab was diluted in 1× PBS to a concentration of 3 μg/mL and 50 μL of Ab was incubated for 16–18 hours at 4°C for coating. Plates were blocked in 1% milk in 1× PBS at 37°C for 1.5 hours. After blocking, plates were washed 4 times with 300 μL of 1× PBST. Samples for mouse total albumin levels were diluted 1:500,000 in 1% milk in 1× PBST. For sample binding, 50 μL of an 8-point standard curve or samples were added to appropriate wells and incubated for 1.5–2 hours on a plate shaker set to 400 rpm. The remainder of the plasma total albumin levels method (detection Ab and developing reagent) is identical to the ALB-2A ELISA protocol.

### Statistics.

Statistical analyses were performed using GraphPad Prism software. Two-tailed *t* test or ANOVA plus Tukey’s or Kruskal-Wallis plus Dunn’s post hoc tests were used as statistical tests for mean comparison. A *P* value less than 0.05 was considered significant. Experimental group sizes are reported in the figures. Data are shown as average ± SEM. Log-rank test was used for survival curve analysis.

### Study approval.

Animal studies were reviewed and approved by the Italian Ministry of Health (authorization 887/2020-PR) and the LogicBio IACUC (authorization LOGC-AUP-02).

### Data availability.

Underlying data for the manuscript can be accessed in the Supporting Data Values XLS file.

## Author contributions

AP designed and performed the experiments in the *Atp7b^–/–^* mouse. MS performed ICP-MS analysis. SC performed liver histological evaluation. EN, FC, and CP performed experiments in the *Atp7b^–/–^* mouse. XBC performed bioinformatic analyses. AB, ME, CCW, and SZ performed experiments in the *Atp7b^tx-J^* mouse. AA supervised ICP-MS analysis. MN designed and supervised studies in the *Atp7b^tx-J^* mouse. PP designed experiments in the *Atp7b^–/–^* mouse, supervised the study, and wrote the manuscript.

## Supplementary Material

Supplemental data

Supporting data values

## Version 1 09/14/2023

In-Press Preview

## Version 2 11/08/2023

Electronic publication
