# Supplementary material for: Genome editing without nucleases confers proliferative advantage to edited hepatocytes and corrects Wilson disease
Source: JCI Insight. 2023 Nov 8;8(21):e171281. doi: 10.1172/jci.insight.171281 (PMC10721260; doi:10.1172/jci.insight.171281)
Supplement: Supplemental data [file jciinsight-8-171281-s131.pdf]

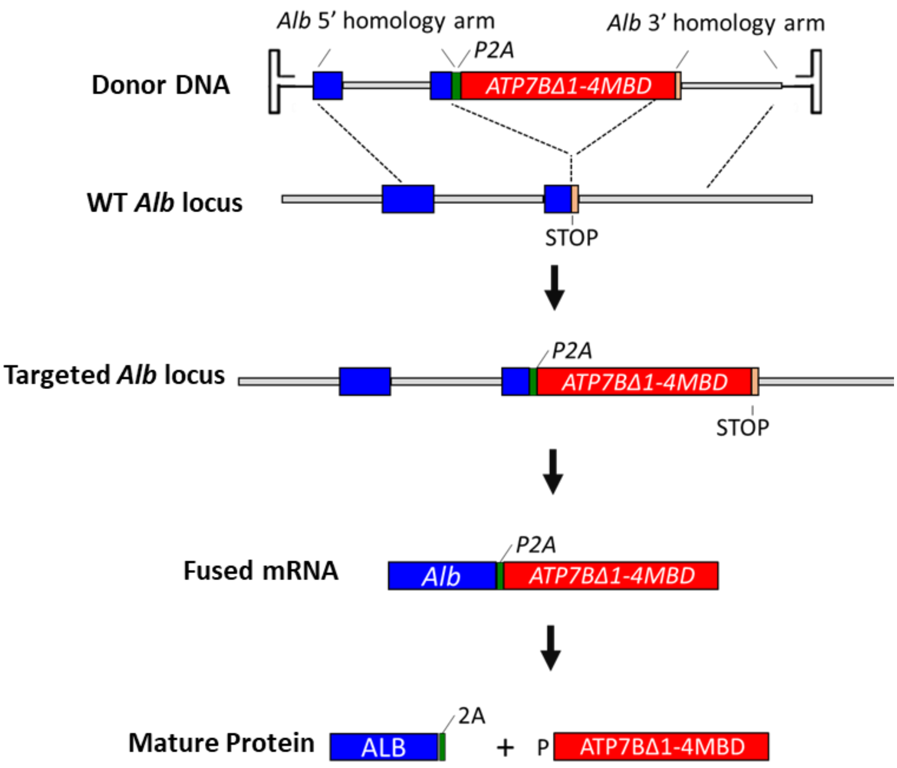

Figure S1. Schematics for the proposed targeting strategy at the *Alb* locus.

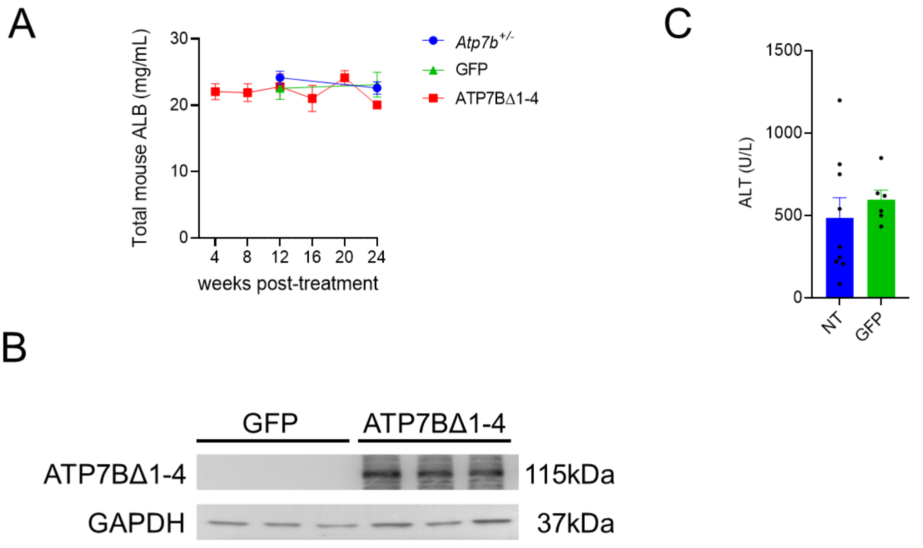

Figure S2. Promoterless mini-ATP7B administration to *Atp7b*$^{-/-}$ mice. 6-week-old *Atp7b*$^{-/-}$ mice were injected with 2.3×10^{13} gc/kg of AAV8-GFP (GFP; n=17) or AAV8-Alb-ATP7BΔ1-4 (ATP7BΔ1-4; n=12). *Atp7b*$^{+/-}$ mice (n=17) are shown as heathy controls. **A)** Total serum albumin levels. **B)** Western blot analysis on whole liver lysates using anti-ATP7B antibody. GAPDH is shown as loading control. **C)** Serum ALT levels in untreated (NT) and AAV8-GFP-injected *Atp7b*$^{-/-}$ mice at 14-18 weeks of age.

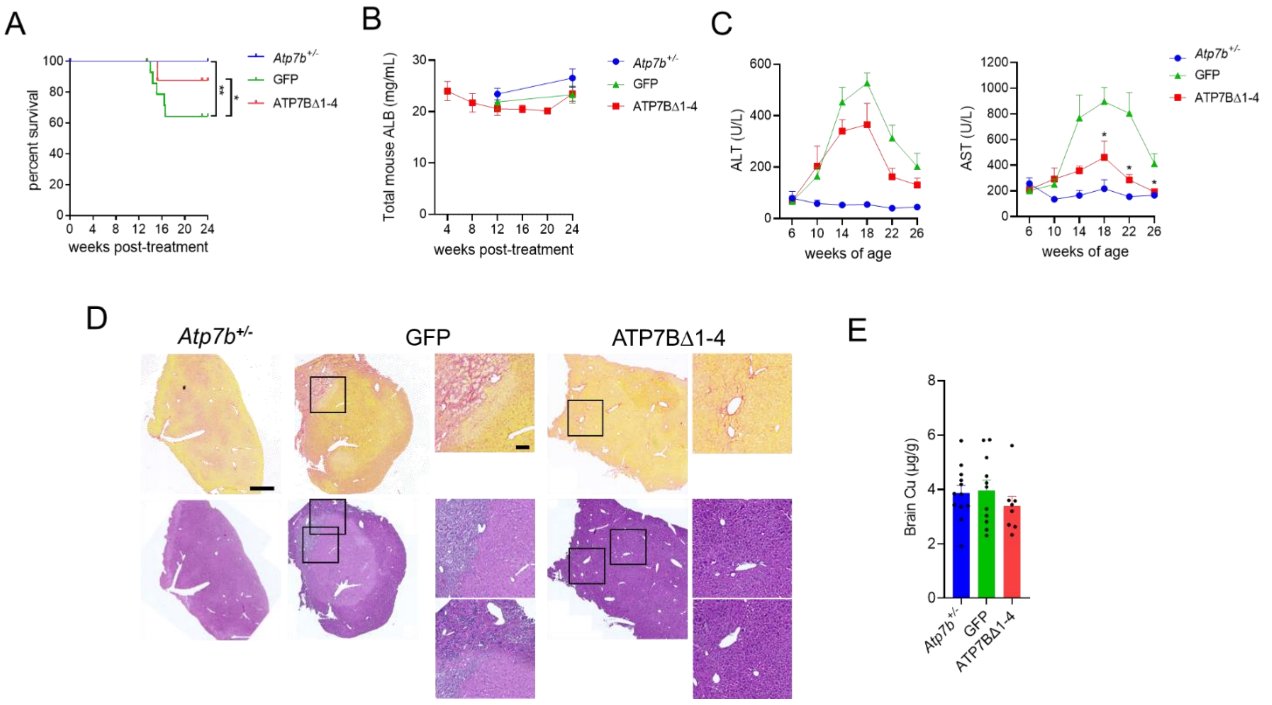

Figure S3. Promoterless mini-ATP7B rescues survival and ameliorates WD. 2-week-old *Atp7b*$^{-/-}$ mice were injected with $2.3x10^{13}$ gc/kg of AAV8-GFP (GFP; n=18) or AAV8-Alb-ATP7BΔ1-4 (ATP7BΔ1-4; n=10). *Atp7b*$^{+/-}$ mice (n=13) are shown as heathy controls. **A)** Survival curves. Log-rank test: *p<0.05; **p<0.01. **B)** Total serum albumin levels. **C)** Serum transaminases levels. Data from animals that survived until the end of the study are shown. Two-way ANOVA plus Tukey's post-hoc: *p<0.05 versus GFP. **D)** Representative images from liver Sirius Red (upper panels) and H&E (lower panels) staining. Scale bar: 200um. **E)** Copper content analysis by ICP-MS in brain.

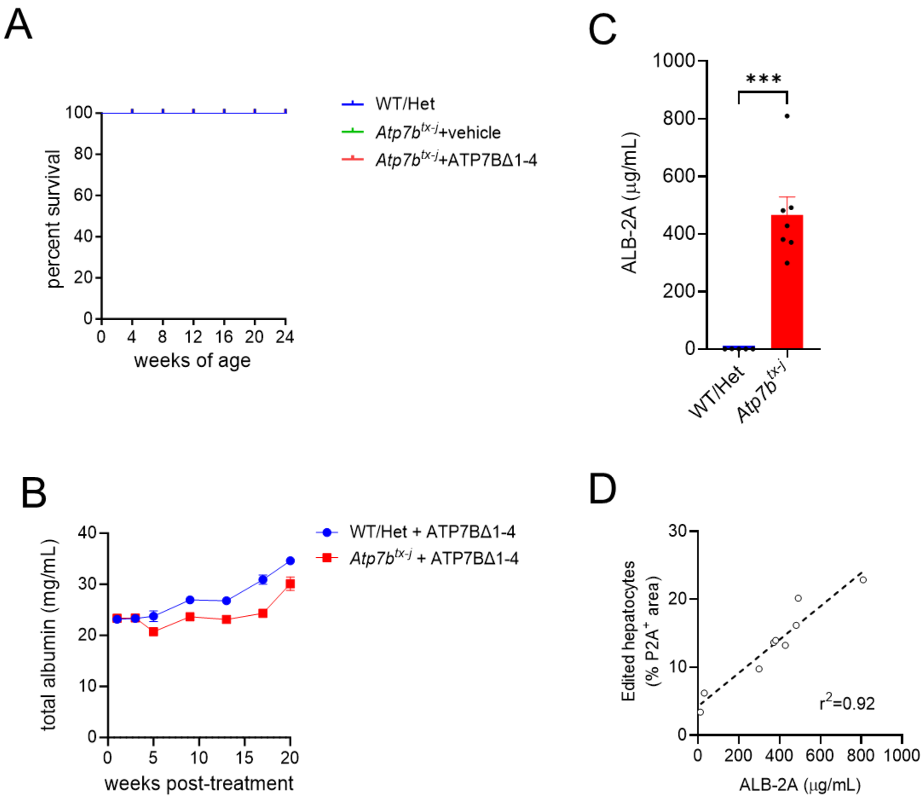

Figure S4. Promoterless mini-ATP7B administration to *Atp7b*$^{tx\text{-}j}$ mice. 3-week-old *Atp7b*$^{tx\text{-}j}$ and healthy control (WT/Het) mice were injected with $1x10^{14}$ vg/kg AAV-DJ-Alb-ATP7BΔ1-4. Tissues were harvested at 25 weeks of age. **A)** Survival curve. Serum **B)** total albumin and **C)** ALB-2A analysis. *t*-test: ***p<0.005 **D)** Linear correlation analysis of P2A staining and serum ALB-2A levels in *Atp7b*$^{tx\text{-}j}$ mice.

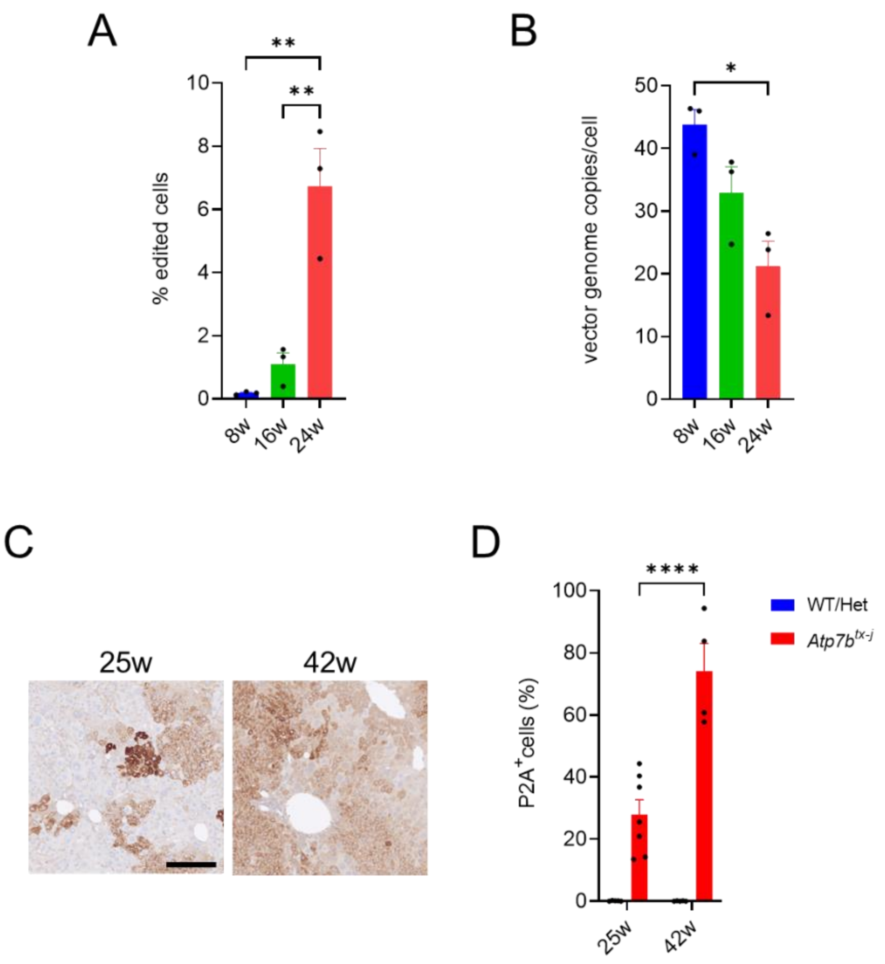

Figure S5: Molecular analysis of mouse liver samples collected over time reveals an expansion of edited hepatocytes. *Atp7b^{tx-j}* mice were dosed 4 weeks after birth with 1x10^{14} vg/kg AAV-DJ-Alb-ATP7BΔ1-4 and a subset of mice were taken down at 8, 16, and 24 weeks after dosing (n=3 per group). **A)** On-target genomic DNA integration analysis. **B)** Assessment of vector genome copy per cell. *Atp7b^{tx-j}* and wild-type or heterozygous (WT/Het) healthy control mice were injected with 1x10^{14} vg/kg AAV-DJ-Alb-ATP7BΔ1-4 3-4 weeks after birth and sacrificed at 25 or 42 weeks of age (n=4-7 per group). **C)** Representative images of immunohistochemical liver staining for P2A. **D)** Quantification of P2A-positive cells. Data are expressed as percentage over total liver cells. One- (A, B) or two-way (D) plus Tukey's post-hoc analysis: *p<0.05; **p<0.01; ****p<0.001

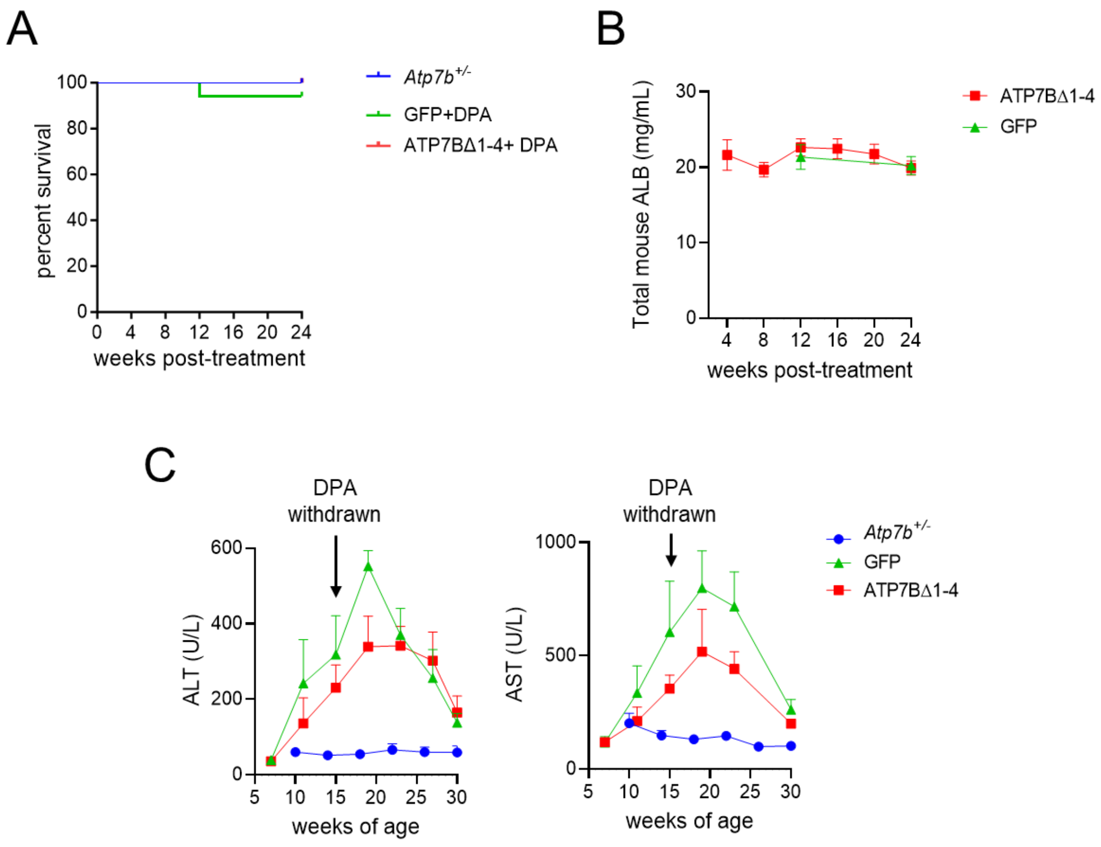

Figure S6. Combination of genome editing and chelator therapy ameliorates WD. 6-week-old *Atp7b*$^{-/-}$ mice were treated with D-penicillamine (DPA) and then injected with 2.3×10^{13} gc/kg of AAV8-GFP (GFP; n=6) or AAV8-Alb-ATP7BΔ1-4 (ATP7BΔ1-4; n=6). *Atp7b*$^{+/-}$ mice (n=13) are shown as heathy controls. **A)** Survival curve. **B)** Total serum albumin levels. **C)** Serum transaminase activities.

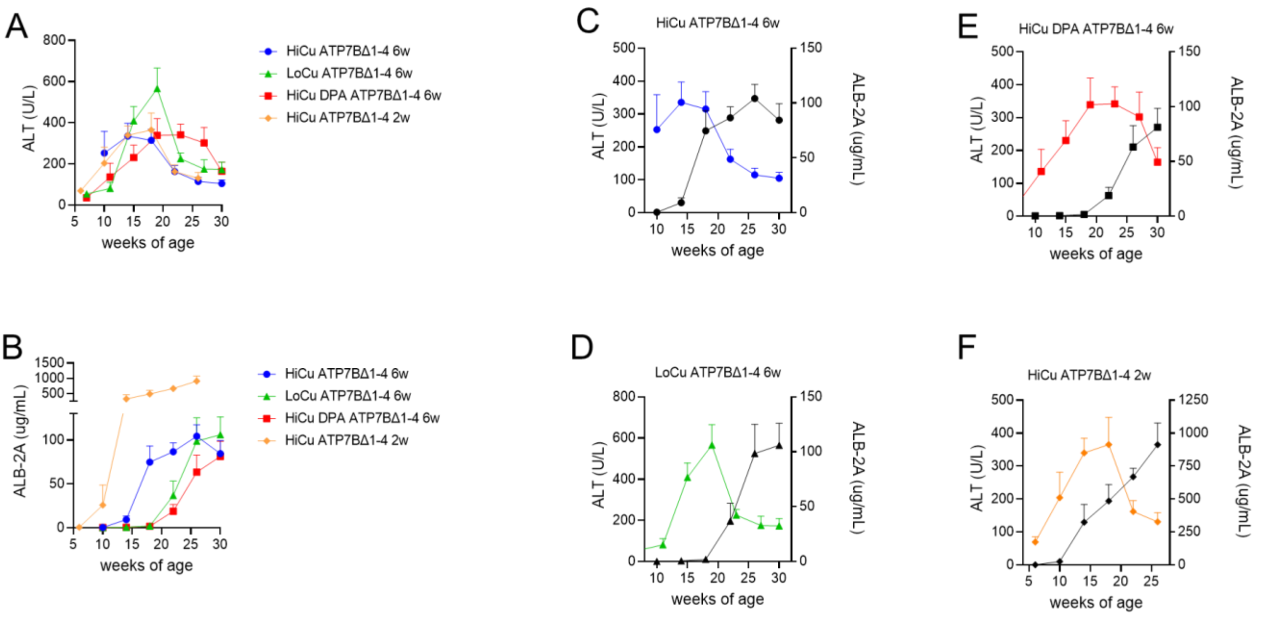

Figure S7. Liver damage modulates repopulation speed by ATP7BΔ1-4-edited hepatocytes in *Atp7b*$^{-/-}$ mice. *Atp7b*$^{-/-}$ mice were fed with high- (HiCu) or low-copper (LoCu) content chow, treated with D-penicillamine (DPA) or left untreated and injected with 2.3x10^{13} gc/kg of AAV8-Alb-ATP7BΔ1-4 at 2 or 6 weeks of age. Serum **A)** ALT and **B)** ALB-2A fusion protein levels in all experimental groups. **C-F)** Serum ALT (left *y* axis, coloured lines) and ALB-2A (right *y* axis, black lines) in single experimental groups. Data from HiCu 6w, HiCu DPA 6w, and HiCu 2w were already presented in previous figures.
